# Complete Genome Sequence of Streptococcus pneumoniae Serotype 19F Strain EF3030

**DOI:** 10.1128/MRA.00198-19

**Published:** 2019-05-09

**Authors:** R. Junges, M. Maienschein-Cline, D. A. Morrison, F. C. Petersen

**Affiliations:** a Institute of Oral Biology, Faculty of Dentistry, University of Oslo, Oslo, Norway; b Research Informatics Core, Research Resources Center, University of Illinois at Chicago, Chicago, Illinois, USA; c Department of Biological Sciences, College of Liberal Arts and Sciences, University of Illinois at Chicago, Chicago, Illinois, USA; University of Maryland School of Medicine

## Abstract

We report the complete genome sequence of Streptococcus pneumoniae EF3030, a serotype 19F isolate that colonizes the nasopharynx of mice while being mostly noninvasive. Such attributes make this strain highly attractive in pneumococcal carriage studies. The availability of its complete genomic sequence is likely to advance studies in the field.

## Abstract (precis)

We report the complete genome sequence of Streptococcus pneumoniae EF3030, a serotype 19F isolate that colonizes the nasopharynx of mice while being mostly noninvasive. Such attributes make this strain highly attractive in pneumococcal carriage studies.

## ANNOUNCEMENT

Streptococcus pneumoniae is the causative agent of important invasive and noninvasive human diseases (1). These morbidities cause a significant financial burden and many mortalities every year, especially in children under 5 years of age (2, 3). The pneumococcal strain EF3030 of the serotype 19F was isolated from a patient with otitis media (4, 5). One of the most interesting features of this strain is its suitability for animal models of pneumococcal disease, as it can colonize the upper respiratory tract for weeks while rarely causing bacteremia (6–10). Such lack of virulence in mice improves the study of host-S. pneumoniae interactions, which are particularly relevant in the assessment of immunization strategies against pneumococcal carriage and disease (11–15). In addition, EF3030 forms dense biofilms and is compatible in models of mixed infection, facilitating an investigation of pneumococcal strain behavior, including the study of natural transformation and other forms of horizontal gene transfer (16).

The S. pneumoniae strain EF3030 was grown in Todd-Hewitt broth supplemented with 5% yeast extract (THY) until late-log phase, and DNA was extracted using the Quick-DNA fungal/bacterial microprep kit (Zymo Research), according to the manufacturer’s protocol. Library preparation utilized the Illumina Nextera XT kit. Sequencing was performed on an Illumina NextSeq platform with 2 × 150-bp reads and an Oxford Nanopore MinION device with a MasterPure complete DNA purification kit (Epicentre, Biosearch Technologies) and the Ligation sequencing kit 1D. Nanopore reads were processed for base calling using Albacore v2.1.10 (17). Reads were assembled using Canu v1.5 (18), which yielded one contig with a total sequence length of 2,142,815 bases. Initial error correction of the Nanopore data assembly was performed using Minimap2 v2.6 (19) and Racon (20), with additional polishing using Illumina data, mapping reads to the contig using BWA-MEM v0.7.15, and fixing single-nucleotide polymorphism (SNP) and indel errors (21). Further analysis of the contig revealed a 41,194-base segment that was duplicated on either end of the contig, which prevented circularization of the genome. To confirm circularization, we trimmed one copy of this duplicated sequence to create a trimmed genome contig of 2,101,618 bases in length. We then remapped reads with BWA-MEM v0.7.15 (21), extracted read pairs where either end mapped within 500 bp of either end of the contig, and performed an assembly on those reads using SPAdes (22). This yielded a single circularization contig of 947 bases in length. Alignment to the trimmed genome contig with nucmer (23) revealed an overlap of the circularization contig with either end of the genome contig, confirming that the trimmed genome contig was circular. We replaced the aligned sequences from the trimmed genome contig with the bases that overlapped with the circularization contig, which added 3 bases to the total genome size for a final circular genome of 2,101,621 bases in length. Finally, we rotated the genome to match the circularized contig to Streptococcus pneumoniae R6 (NCBI RefSeq accession no. NC_003098) with nucmer (23), and the start position of the genome was set as the origin of replication locus upstream of the *dnaA* gene (24). Gene annotation was performed following the NCBI Prokaryotic Genome Annotation Pipeline revision 4.7 (25).

The complete genome of S. pneumoniae EF3030 presents a GC content of 39.8% and 2,222 genes, including 2,149 coding sequences (CDS), 73 RNA-coding genes (4 complete rRNA operons, 58 tRNAs, and 3 noncoding RNAs [ncRNAs]), and 210 pseudogenes. The hybrid assembly filled the 85 gaps in the recent EF3030 draft genome (26). Newly identified genes included the capsule locus that contains the genes specific to the strain EF3030 serotype 19F (capsule locus starts at position ∼306000). Among these are the glycosyltransferase genes *wchO* (EF3030_01700), *wchP* (EF3030_01705), and *wchQ* (EF3030_01710), the polymerase gene *wzy* (EF3030_01715), the flippase gen *wzx* (EF3030_01720), and the *mnaA* and rhamnose pathway genes *rmlABCD* (EF3030_01725 to EF3030_01745). Altogether, we expect that the availability of the complete genome sequence for S. pneumoniae EF3030 will facilitate the genetic manipulation of this strain and the further study of pneumococcal colonization and disease.

### Data availability.

The nucleotide sequence of the S. pneumoniae EF3030 genome is deposited in NCBI GenBank under accession no. CP035897, and the raw reads are available in the Sequence Read Archive with BioProject no. PRJNA521678.
